# Road to achieving COVID‐19 herd immunity in Niger Republic: Challenges and recommendations

**DOI:** 10.1002/puh2.38

**Published:** 2022-12-07

**Authors:** Sil‐Ana Salissou Abdou, Oladunni Abimbola Amos, Ogechi Vida Onwuemene, Goodness Ogeyi Odey, Caleb James Nsikak Abasi

**Affiliations:** ^1^ Faculty of Health Sciences, department of Nursing and midwifery Aboubacar Ibrahim International University Maradi Maradi Niger Republic; ^2^ Department of Pharmacology and Therapeutics College of Medicine University of Ibadan, Ibadan, Oyo state Nigeria; ^3^ Afe Babalola Multisystem Hospital, Ado‐Ekiti, Ekiti state Nigeria; ^4^ Department of Public Health University of Calabar Calabar Nigeria; ^5^ Faculty of Pharmacy University of Uyo, Uyo, Akwa Ibom Nigeria

**Keywords:** COVID‐19, herd immunity, Niger Republic, vaccine

## Abstract

COVID‐19 pandemic has had tremendous impact on countries across the world and Niger Republic is not left out of the ravaging impact of the virus. The rapid dissemination of the virus across the globe has led to the development of safe and efficacious vaccines at an unprecedented level. While Niger Republic has prioritized COVID‐19 vaccination in line with the global plan to attain herd immunity by vaccinating 70% of world's population, the country has continued to struggle to expand coverage of its population. Niger Republic is faced with challenges such as conflict, COVID‐19 vaccine hesitancy, poor COVID‐19 vaccine demand and inefficient information system, consequently leading to low COVID‐19 vaccine demand and uptake. These challenges coupled with the different wave of the pandemic, the latest largely driven by the Omicron variant has slowed down progress towards achieving herd immunity in Niger Republic. There is need for the Nigerien government to scale up vaccination drive as well as implement refined approaches towards achieving the country's herd immunity target. This paper aimed to discuss the current state of COVID‐19 vaccination including efforts and challenges towards achieving herd immunity in Niger Republic, thus informing strategies to ramp up COVID‐19 vaccination in the country.

## INTRODUCTION

Coronavirus Disease 2019, also known as COVID‐19 has had significant impact on all countries worldwide and Niger Republic is not left out of the devastating impact of the virus. Niger Republic is a landlocked western African country covering 1,276,000 km^2^ [1]. The country reported its first case of COVID‐19 in Niamey, the capital city of Niger Republic on March 19, 2020. The case was a 36 year old Nigerien returning from Togo [2]. With the high rate of migration of populations, the virus became rapidly distributed throughout the country. This prompted the Government to implement several measures to limit the spread of the virus including land border closure and flight suspension [2]. Several policies were also put in place to train more healthcare workers, hygiene technicians and laboratory technicians in infection prevention and control measures, GeneXpert application and psychosocial care protocols [3]. In addition, the COVID‐19 emergency response project, an initiative funded by the World Bank through the International Development Association (IDA) assisted the country via medication procurement, supply of personal protective equipment and emergency fund transfer in order to alleviate the health and economic effect of the pandemic [4]. While the Nigerien government has remained undeterred by putting in place several measures to mitigate the impact of the pandemic on the health system and economy, the existing burden of insecurity and environmental shocks have continue to undermine the country's efforts. As of March 9, 2022, approximately 8769 cases and 307 deaths associated with COVID‐19 were reported in Niger Republic [5].

In response to the high rate of morbidity and mortality worldwide, collaborative efforts of scientists, governments and civil society organizations to produce COVID‐19 vaccines have yielded tremendous results at an unprecedented level [6]. In line with this, the WHO in collaboration with relevant stakeholders established guidelines to deliver vaccines to all countries worldwide [6]. Niger Republic received its first COVID‐19 vaccine delivery on April 14, 2021 through COVID‐19 Vaccine Global Alliance (COVAX) facility, the Global Alliance for equitable access to vaccines [7]. As of February 26 2022, 5.87% of eligible populations have received at least one dose of COVID‐19 vaccine. Although Niger Republic has shown commitment to protect vulnerable populations through national COVID‐19 vaccination drive, challenges such as limited fund and vaccine hesitancy has undermine the efforts towards achieving herd immunity in the country. In this paper we discuss the current state of vaccination and challenges faced including strategies that can be adapted to ramp up COVID‐19 vaccination in Niger Republic.

## COVID‐19 IMPACT ON HEALTHCARE AND ECONOMY

The introduction of COVID‐19 in Niger Republic has put additional pressure on an existing weakened healthcare system that is characterized by limited health resource, limited healthcare coverage, poor health insurance coverage and limited funding. With rapid increase in the number of cases, there are limited hospital beds to accommodate patients [8]. Also, Inadequate infection prevention and control measures in hospitals coupled with lack definite isolation units and lack of staff training has led to increased COVID‐19 exposure risk among healthcare workers at integrated health centers and private clinics [9]. With healthcare workers reportedly constituting approximately 17% of total COVID‐19 cases in Niger Republic, additional pressure has been mounted on the country's health care system [9].

COVID‐19 has had significant impact on Niger's economy including livelihood of the populations. During the heat of the pandemic in 2020, the imposed lockdown measures and movement restrictions caused economic shutdown with significant job losses across the country, pushing more than 370,000 people into extreme poverty. Also, approximately 0.3% decline in per capital income was recorded, causing 1.3% increase in proportion of population living on less than 1.9 USD per day [10]. This situation is complicated by series of security crises and floods, causing displacement of people from their homes and approximately 90, 000,000 USD losses in the agricultural sector of the country [10].

**FIGURE 1:**
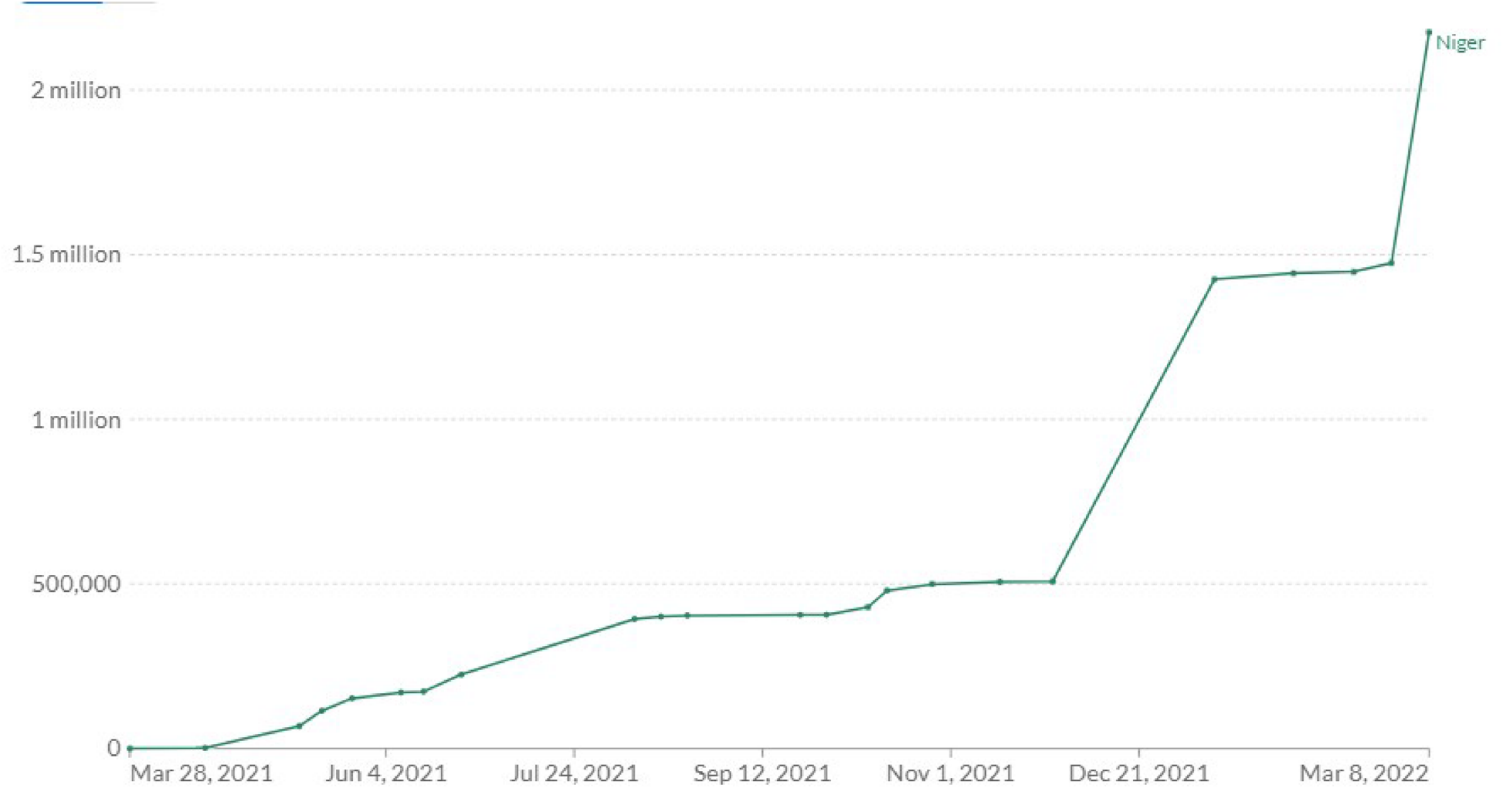
Share of people who have been partially vaccinated. *Source*: Our World in Data

**FIGURE 2:**
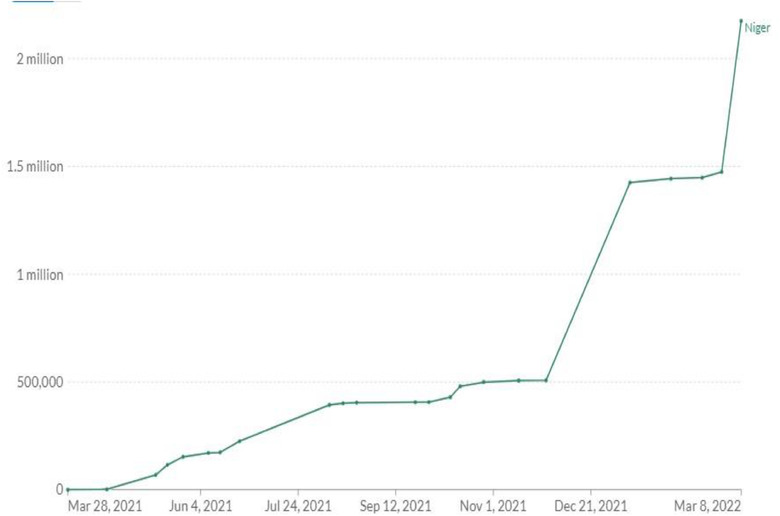
Share of people who have been fully vaccinated. *Source*: Our World in Data

**COVID‐19 Vaccination** In line with global drive to vaccinate populations against COVID‐19, Niger Republic has prioritized COVID‐19 vaccination as a crucial step to drive down transmission and protect at risk populations [11]. The country began vaccination campaign in March 2021 [12] following the delivery of the China Sinopharm Vaccine [13]. Healthcare workers, teachers, defense personnel, refugees and vulnerable populations were identified as priority groups to receive COVID‐19 vaccines. In April 2021, the country received the first batch of COVID‐19 vaccines delivered through COVAX facility (Frank, 2021a). Other vaccines approved for use in Niger Republic include the Johnson and Johnson vaccine, AstraZeneca vaccine and Pfizer vaccine [14]. As of March 8, 2022, approximately 8% of eligible populations in Niger Republic have received at least one dose of COVID‐19 vaccine while 6.4% have been fully vaccinated [15] (Figures 1 and 2).

## CHALLENGES TO ATTAINING HERD IMMUNITY

As Niger Republic drive vaccination campaign against COVID‐19, the country has continued to face challenges such as conflict and violent, COVID‐19 vaccine hesitancy, poor COVID‐19 vaccine demand and inefficient information system. Prior to the pandemic, insecurity crisis in Niger republic is characterized by the Central Sahel Crisis in the Western region, the Lake Chad Basin Crisis in southeastern region, banditry and activities of non‐state harmed groups in southwestern region [16]. This situation has led to the displacement of people from their homes, posing significant threat to public health. As a result, these populations are at risk of missing out on COVID‐19 vaccine coverage, thereby slowing down progress toward achieving herd immunity in Niger republic.

Vaccine hesitancy fueled by mistrust in existing structure has significantly contributed to low vaccine demand and uptake. According to a study conducted in Niger Republic in 2021, an estimated 56% of people had little or no trust in the Government ability to ensure development and delivery of safe COVID‐19 vaccines [17]. Consequently, this has created a sub‐population of unimmunized individuals among people who are eligible to receive COVID‐19 vaccines.

Low COVID‐19 vaccine demand has led to low uptake which varies across geographic and socioeconomic level. An estimated 58% of people surveyed in Niger Republic are unlikely to receive COVID‐19 vaccines [17]. Several factor such as belief system, income status and educational status have been identified to be associated with poor COVID‐19 vaccine demand and uptake [17], leading to limited COVID‐19 vaccination coverage. Significant proportion of respondents (89%) believed prayer to be more effective than vaccine in preventing COVID‐19 [17, 21].There is need to engage and mobilize religious leaders, who can prove to be one of the strong allies in expanding COVID‐19 vaccination coverage in Niger Republic.

COVID‐19 vaccine information gap across educational and socio‐economic groups has created misinformation and poor vaccine acceptance, thus slowing down progress in achieving herd immunity. An estimated 57% of populations living in poverty and 53% of populations with no formal educations expressed unwillingness to receive COVID‐19 jab [17]. This situation impedes readiness to accept COVID‐19 vaccines and constitutes a major obstacle to the prevention and control of the pandemic across Niger Republic. More effort is needed through public health campaign to reinforce confidence and willingness to vaccinate populations with lower educational status and populations with lower income status who are uncertain or unwilling to take‐up vaccines. With the emergent of resistant strains of COVID‐19, the latest been the Omicron variant, this presents serious challenge to COVID‐19 prevention and control including progress towards attaining herd immunity in Niger Republic.

## RECOMMENDATIONS

### Military involvement and geographic intelligent report

The importance of engaging the military in vaccination coverage in violent and conflict affected areas cannot be overemphasized. The Nigerien Government should develop innovative mechanisms to deliver COVID‐19 vaccine to security‐inaccessible areas in the country. This can be achieved through specialized orientation and training of military personnel in vaccination protocols, military support in vaccination activities and provision of local intelligent reports. Military geographic intelligence report can provide the vaccination team with field protection and security tips to predetermine initial safe storage sites for COVID‐19 vaccines from where it can be dispatched to security‐inaccessible areas as well as identify and prioritize at‐risk populations. Knowledge of geospatial data can help provide information on gaps in vaccination coverage, thus informing approaches to formulate alternative vaccine distribution protocols.

### Engaging the community

Negative perception and misinformation on vaccine can potentially affect people's attitude, acceptance and uptake of COVID‐19 vaccines [18]. Lack of trust in the safety and effectiveness may fuel COVID‐19 vaccine delay and refusal [19]. The WHO recommends that countries should implement context specific strategies that increase vaccine acceptance and demand [20]. A robust public engagement in COVID‐19 vaccine roll out is crucial in overcoming lack of trust and increasing demand and uptake in Niger Republic. Although the country has taken important step by engaging traditional and community leaders against looming COVID‐19 vaccine hesitancy [21], there is need to strengthen effective communication with members of the community. This will help to revive community trust and facilitate transparent communication between community leaders and residents, thus promoting positive vaccine behaviors. In addition, there is need to engage communities through the integration of local knowledge, information sharing, training of residents as vaccinators and building community mobilization networks. Specific activities relating to vaccination such as collecting and analyzing data on public perception, feedback, and belief should be integrated into COVID‐19 vaccination strategies. This approach will help to understand perception of people about COVID‐19 and to identify specific community concerns, providing evidences for targeted approaches to increasing COVID‐19 vaccine demand and uptake.

### Aggressive and safe COVID‐19 vaccine rollout

Niger Republic faces an unprecedented response to COVID‐19 pandemic in a period with an underdeveloped and underfunded healthcare system [22]. The country must set daily COVID‐19 vaccination targets as well as develop and implement refined approaches to achieving these targets. This will help intensify vaccination of the population including high‐risk groups. There is need for Niger Republic to adapt vaccination strategies to community needs through a tailored and targeted mass vaccination campaign in order to achieve herd immunity.

Additional funding will enable Niger Republic to procure and distribute COVID‐19 vaccines, strengthen relevant health systems necessary for successful vaccination campaign and prepare for future pandemics [12]. It is crucial for the country to increase funding and strengthen commitment to COVID‐19 vaccine delivery actions, thereby ensuring that the country has the required financial resources to set in motion its vaccination drive and to increase access to COVID‐19 vaccines. Niger Republic has taken an important step in the fight against the pandemic by implementing adaptive solution through community‐based surveillance system (CBS) for passive case identification [23]. However, as the country evolves from its fourth pandemic wave, the government must scale up disease surveillance, infection prevention and control measures, training of community vaccinators and support staffs and ensure efficient COVID‐19 storage capacity across the country.

## CONCLUSION

With global commitment to vaccinate 70% of the world's population with COVID‐19 vaccines by mid 2022, African countries must strengthen commitments to ram up vaccination drive. While COVID‐19 vaccine delivery to the continent has significantly increased, many countries are struggling to expand coverage. There is a need for the Nigerien government to scale up vaccination drive as well as implement refined approaches towards achieving the country's herd immunity target.

## AUTHOR CONTRIBUTIONS

Sil‐Ana Salissou Abdou: Conceptualization; Data curation; Formal analysis; Investigation; Methodology; Project administration; Resources; Supervision; Validation; Visualization; Writing‐original draft; Writing‐review & editing. Ogechi Vida Onwuemene: Conceptualization; Data curation; Investigation; Methodology; Resources; Writing‐original draft. Goodness Ogeyi Odey: Investigation; Methodology; Writing‐original draft; Writing‐review & editing.

## CONFLICT OF INTEREST

Dr. Goodness Ogeyi Odey is a Youth Editorial Board member of Public Health Challenges and a co‐author of this article. To minimize bias, she has been excluded from all editorial decision‐making related to the acceptance of this article for publication.

## Data Availability

All data relevant to the study are included in the article.
